# Abnormal cortical synaptic transmission in Ca_V_2.1 knockin mice with the S218L missense mutation which causes a severe familial hemiplegic migraine syndrome in humans

**DOI:** 10.3389/fncel.2015.00008

**Published:** 2015-02-17

**Authors:** Dania Vecchia, Angelita Tottene, Arn M.J.M. van den Maagdenberg, Daniela Pietrobon

**Affiliations:** ^1^Department of Biomedical Sciences, University of Padova, and Consiglio Nazionale delle Ricerche (CNR) Institute of NeurosciencePadova, Italy; ^2^Departments of Human Genetics and Neurology, Leiden University Medical CentreLeiden, Netherlands

**Keywords:** calcium channel, synaptic transmission, cortex, migraine, spreading depression, excitatory, inhibitory, knockin mouse

## Abstract

Familial hemiplegic migraine type 1 (FHM1) is caused by gain-of-function mutations in Ca_V_2.1 (P/Q-type) Ca^2+^ channels. Knockin (KI) mice carrying the FHM1 R192Q missense mutation show enhanced cortical excitatory synaptic transmission at pyramidal cell synapses but unaltered cortical inhibitory neurotransmission at fast-spiking interneuron synapses. Enhanced cortical glutamate release was shown to cause the facilitation of cortical spreading depression (CSD) in R192Q KI mice. It, however, remains unknown how other FHM1 mutations affect cortical synaptic transmission. Here, we studied neurotransmission in cortical neurons in microculture from KI mice carrying the S218L mutation, which causes a severe FHM syndrome in humans and an allele-dosage dependent facilitation of experimental CSD in KI mice, which is larger than that caused by the R192Q mutation. We show gain-of-function of excitatory neurotransmission, due to increased action-potential evoked Ca^2+^ influx and increased probability of glutamate release at pyramidal cell synapses, but unaltered inhibitory neurotransmission at multipolar interneuron synapses in S218L KI mice. In contrast with the larger gain-of-function of neuronal Ca_V_2.1 current in homozygous than heterozygous S218L KI mice, the gain-of-function of evoked glutamate release, the paired-pulse ratio and the Ca^2+^ dependence of the excitatory postsynaptic current were similar in homozygous and heterozygous S218L KI mice, suggesting compensatory changes in the homozygous mice. Furthermore, we reveal a unique feature of S218L KI cortical synapses which is the presence of a fraction of mutant Ca_V_2.1 channels being open at resting potential. Our data suggest that, while the gain-of-function of evoked glutamate release may explain the facilitation of CSD in heterozygous S218L KI mice, the further facilitation of CSD in homozygous S218L KI mice is due to other Ca_V_2.1-dependent mechanisms, that likely include Ca^2+^ influx at voltages sub-threshold for action potential generation.

## Introduction

Ca_V_2.1 (P/Q-type) Ca^2+^ channels play a prominent role in initiating action potential (AP)-evoked neurotransmitter release at brain excitatory and inhibitory synapses (Pietrobon, 2005, 2010). Several neurological disorders including familial hemiplegic migraine type 1 (FHM1), a rare monogenic form of migraine with aura, are caused by mutations in the *CACNA1A* gene, which encodes for the α_1A_ pore-forming subunit of Ca_V_2.1 channels (Ophoff et al., 1996; de Vries et al., 2009; Pietrobon, 2010). Migraine is a common brain disorder whose key manifestation is the recurrence of attacks of unilateral headache due to activation of the trigeminovascular pain pathway (Pietrobon and Moskowitz, 2013). There is compelling evidence that headache mechanisms can be triggered by cortical spreading depression (CSD), the phenomenon that underlies migraine aura (Noseda and Burstein, 2013; Pietrobon and Moskowitz, 2013, 2014). The mechanisms of primary brain dysfunction that lead to the onset of a migraine attack and to increased susceptibility to CSD, however, remain a major open issue. Insights into these mechanisms can be obtained from the functional analysis of transgenic knockin (KI) mice carrying FHM1 missense mutations that were introduced into the orthologous *Cacna1a* gene. FHM1 KI mice were shown to exhibit a lower threshold for induction of experimental CSD and a higher velocity of CSD propagation compared with wild-type (WT) mice (van den Maagdenberg et al., 2004, 2010).

FHM1 mutations produce gain-of-function of single human recombinant Ca_V_2.1 channels, mainly due to channel activation at lower voltages and increased channel open probability (Tottene et al., 2002, 2005; Pietrobon, 2013). Accordingly, a larger P/Q-type Ca^2+^ current density in a broad range of mild depolarizations was measured in several types of neurons (including cortical pyramidal cells) from KI mice that carry either the FHM1 R192Q or the S218L mutation (van den Maagdenberg et al., 2004, 2010; Tottene et al., 2009; Inchauspe et al., 2010; Fioretti et al., 2011; Gao et al., 2012; Di Guilmi et al., 2014). Whereas mutation R192Q in humans causes pure FHM (Ophoff et al., 1996), mutation S218L is associated with a severe clinical syndrome, that may consist of cerebellar ataxia, seizures, coma and sometimes fatal cerebral edema triggered by a trivial head trauma, in addition to FHM (Kors et al., 2001; Stam et al., 2009). In accordance with the more severe clinical phenotype, the S218L mutation produces: (i) a larger shift to lower voltages of activation of human Ca_V_2.1 channels than the R192Q mutation (Hans et al., 1999; Tottene et al., 2005; Pietrobon, 2010); (ii) a larger gain-of-function of the P/Q-type Ca^2+^ current at low voltages in neurons of homozygous S218L (SL/SL) compared with homozygous R192Q (RQ/RQ) KI mice (van den Maagdenberg et al., 2004, 2010; Inchauspe et al., 2010; Di Guilmi et al., 2014); and (iii) a larger facilitation of induction and propagation of experimental CSD in SL/SL compared with RQ/RQ KI mice *in vivo* (Eikermann-Haerter et al., 2009, 2011; van den Maagdenberg et al., 2010). Both the gain-of-function of the neuronal P/Q-type Ca^2+^ current and the facilitation of experimental CSD in heterozygous S218L (SL/WT) KI mice were similar to those in RQ/RQ KI mice (van den Maagdenberg et al., 2010).

Investigation of cortical synaptic transmission in RQ/RQ KI mice revealed an increased strength of excitatory neurotrans-mission at cortical pyramidal cell synapses, due to increased probability of glutamate release consequent to increased AP-evoked Ca^2+^ influx through presynaptic P/Q-type Ca^2+^ channels compared with those at WT synapses (Tottene et al., 2009). In striking contrast, inhibitory synaptic transmission at fast-spiking and other multipolar interneuron synapses was unaltered, due to unaltered AP-evoked Ca^2+^ influx and GABA release (Tottene et al., 2009; Vecchia et al., 2014). Restoration of AP-evoked glutamate release at cortical pyramidal cell synapses of RQ/RQ KI mice to the WT value completely eliminated the facilitation of experimental CSD, thus supporting a causative link between enhanced glutamate release and facilitation of CSD in FHM1 KI mice (Tottene et al., 2009). The differential effect of the R192Q mutation at cortical excitatory and inhibitory synapses suggests altered regulation of the cortical excitatory-inhibitory balance in FHM1 and points to a disruption of this balance and neuronal network hyperactivity as the basis for an episodic vulnerability to CSD ignition in migraine (Tottene et al., 2009; Vecchia and Pietrobon, 2012).

The effect of other FHM1 mutations, including the more severe S218L mutation, on cortical synaptic transmission remains unknown. Here, we studied excitatory and inhibitory neurotransmission in microcultures of cortical neurons from neonatal S218L KI and WT mice to investigate: (i) whether the S218L mutation, like the R192Q mutation, enhances excitatory neurotransmission by increasing AP-evoked Ca^2+^ influx and glutamate release at cortical pyramidal cell synapses, but does not affect inhibitory neurotransmission at multipolar interneuron synapses; (ii) whether the gain-of-function of evoked glutamate release in SL/WT and SL/SL KI mice correlates with the similar gain-of-function of the P/Q-type Ca^2+^ current and facilitation of CSD in SL/WT and RQ/RQ KI mice and with the larger gain-of-function of the P/Q-type Ca^2+^ current and facilitation of CSD in SL/SL than SL/WT and RQ/RQ KI mice; and (iii) whether, as a consequence of the particularly low threshold of activation of mutant S218L channels, a fraction of these channels are open at resting membrane potential in cortical glutamatergic synaptic terminals.

## Materials and methods

### Preparation of cortical neurons in microculture

Knockin mice carrying the S218L mutation in the *Cacna1a* gene were generated as previously described (van den Maagdenberg et al., 2010). Cortical neurons were isolated from P0-2 homozygous SL/SL or heterozygous SL/WT KI mice and WT mice with the same genetic background following the procedure of Levi et al. (1984). The neurons were cultured on glial microislands (at the density of 6,000–25,000 cells/mL) essentially as reported in Brody and Yue (2000) for hippocampal neurons, except for the following details: astrocytes culture medium was Basal Eagle’s Medium (BME) plus 10% fetal bovine serum, 25 mM KCl, 2 mM glutamine and 50 µg/mL gentamicin; neuronal medium was Neurobasal A plus 2% B27 Supplement, 0.5 mM glutamine and 1% PSN Antibiotic mix (all from Gibco); only half of the volume of the astrocytes medium was replaced with neuronal medium to allow the astrocytes to condition the medium before neuron plating. Every 4 days half of the volume of neuronal medium was refreshed.

Single cortical neurons grown on glial microislands form synaptic connections onto themselves (autapses) with properties very similar to those of synapses between neurons; these autaptic connections are by definition monosynaptic, offer an unusually homogeneous population of synapses producing large synaptic responses and solution exchanges can be fast and complete (Bekkers and Stevens, 1991).

All experimental procedures were carried out in accordance with the Italian Animal Welfare Act and with the Use Committee guidelines of the University of Padova and were approved by the local authority veterinary service.

### Electrophysiological recordings and data analysis

Whole-cell patch-clamp recordings were made at room temperature following standard techniques. Electrical signals were recorded through an Axopatch-200B or Multiclamp 700B patch-clamp amplifier and digitized using a Digidata 1440A or Digidata 1322A interface and pClamp software (Axon Instruments).

Microislands containing several glial cells and a single neuron were selected for recording of evoked postsynaptic currents (PSCs) in voltage-clamp mode (sampling 5 KHz; filter 1 KHz) after 8–14 days (DIVs) in culture. APs in the unclamped processes were induced by a 2 ms voltage pulse to +20 mV every 10 s from a holding potential of −80 mV. The evoked PSCs were measured at −80 mV and −90 mV for excitatory and inhibitory PSC, respectively. Single neurons with a pyramidal cell morphology characterized by a typical prominent process emanating from a triangular/pyramidal soma were selected for recording glutamate receptor-mediated excitatory postsynaptic currents (EPSCs), as in Tottene et al. (2009). The currents recorded in the presence of 5 µM NBQX were subtracted to all records to obtain the evoked EPSCs (displayed in the figures after blanking 1–2 ms around each stimulus artifact for clarity). Single neurons with irregular soma morphology and multiple asymmetrical processes emanating from it (multipolar interneurons) were selected for recording inhibitory postsynaptic currents (IPSCs), as described in Vecchia et al. (2014). Although the GABA_A_-mediated IPSCs recorded at −90 mV are inward currents (given the predicted and measured E_rev_ of −69 and −63 mV, respectively), they were easily distinguished from glutamate receptor-mediated EPSCs on the basis of their slower time course and their complete inhibition by 20 µM bicuculline (Ascent Scientific-Abcam). The currents recorded in the presence of bicuculline were subtracted to all records to obtain the evoked IPSCs (displayed in the figures after blanking 1–2 ms around each stimulus artifact for clarity). After PSC stabilization (typically 3 min after break-in), 5–10 sweeps were averaged to obtain the PSC amplitude.

The pipette solution contained (in mM): 110 K-methane-sulfonate, 5 MgCl_2_, 30 HEPES, 3 EGTA, 4 ATP, 0.5 GTP and 1 cAMP (pH 7.4 with KOH). The extracellular solution contained (in mM): 145 NaCl, 3 KCl, 10 HEPES, 10 glucose, 1 MgCl_2_, 2 CaCl_2_, 0.05 D-AP5 (pH 7.4 with NaOH). In the experiments testing the effect of the peptide toxin ω-AgaIVA (Aga) (Peptide Institute Inc.), cytochrome C (0.1 mg/mL) was added to the solution.

A liquid junction potential (LJP) of −8 mV should be added to all voltages to obtain the correct membrane potentials (Neher, 1992). Patch-clamp pipettes had resistances of 1.8–2.5 MΩ. Compensation (typically 60–80%) for series resistance was used (2.5–6 MΩ after compensation).

The relative number of neurons recorded from WT and KI mice at different DIVs was closely matched.

Acute coronal slices of the barrel cortex were prepared from P16-18 mice as described in Tottene et al. (2009). Miniature EPSCs (mEPSCs) were recorded from layer 2/3 pyramidal cells that were identified based on their morphology and firing pattern in response to current injection as in Tottene et al. (2009). The pipette solution contained (in mM): 6 KCl, 114 K-gluconate, 10 HEPES, 10 phosphocreatine, 4 MgATP, 0.3 NaGTP (pH 7.25 with KOH, osmolarity 300 mOsm with sucrose. Slice were superfused with a modified ACSF containing (in mM): 125 NaCl, 3.5 KCl, 25 NaHCO_3_, 1.25 NaH_2_PO_4_, 0.5 MgCl_2_, 1 CaCl_2_, 25 glucose saturated with 95% O_2_ 5% CO_2_. In order to record mEPSCs, after the break-in and the recording of the firing pattern, slices were superfused with the same ACSF solution containing (in µM): TTX 0.2, D-AP5 50, bicuculline 20 and cytochrome C (0.1 mg/mL). mEPSCs were acquired at −69 mV for at least 3 min (sampling 10 KHz; filter 2 KHz) before and after application of 400 nM Aga and analyzed using Clampfit 10.0 (Axon Instruments), as in Tottene et al. (2009). Series resistance was not compensated (Rs was below 25 MΩ with less than 20% variation); LJP −12 mV.

Data are given as mean ± SEM; stars indicate a statistically significant difference from control assessed by the unpaired or paired Student’s *t* test (*, *p* < 0.05; **, *p* < 0.01; ***, *p* < 0.001).

## Results

**Figure 1:**
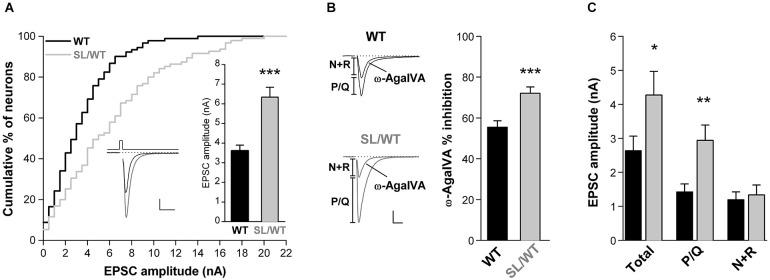
**Increased EPSC amplitude and increased contribution of P/Q-type Ca^2+^ channels to excitatory neurotransmission in cortical pyramidal cell autapses of SL/WT KI mice. (A)** Cumulative distribution and average value (right inset) of EPSC amplitudes evoked in single WT and SL/WT cortical pyramidal cells in microculture for 10 to 14 days (DIV). EPSC amplitude: 6.3 ± 0.5 nA (*n* = 95) in SL/WT and 3.6 ± 0.3 nA (*n* = 91) in WT neurons (*t*-test: *p* = 5.0 × 10^−6^). Left inset: representative EPSC traces from a WT (black) and a SL/WT (gray) neuron; scale bars 10 ms, 1 nA. The relative number of neurons recorded from WT and KI mice at different DIV were closely matched, because EPSC amplitude continued to increase from 10 to 14 DIV in both control and SL/WT neurons (Tottene et al., 2009). **(B)** Contribution of P/Q-type Ca^2+^ channels to excitatory neurotransmission evaluated from the fraction of the EPSC inhibited by Aga (200 nM, a saturating concentration since the EPSC was not further inhibited by 400 nM Aga (Tottene et al., 2009)) in cortical pyramidal cells in microculture (DIV 8–12): 72 ± 3% (*n* = 19) in SL/WT and 56 ± 3% (*n* = 21) in WT neurons (*t*-test: *p* = 6.1 × 10^−4^). Representative EPSC traces before and after Aga are shown on the left; scale bars: 10 ms, 1 nA. The relative number of neurons recorded from WT and KI mice at different DIV were closely matched. **(C)** Average values of the amplitudes of the EPSC (total) and its Aga-sensitive (P/Q) and Aga-insensitive (N+R) components (same neurons as in **B**). The P/Q and N+R components for each cell were obtained (as shown in the representative traces in **B**) as difference of EPSC amplitude before and after Aga (P/Q) and the EPSC amplitude remaining after Aga (N+R). Values of EPSC amplitudes: 4.3 ± 0.7 in SL/WT vs. 2.6 ± 0.4 in WT (Total; *t*-test: *p* = 0.048); 2.9 ± 0.5 in SL/WT vs. 1.4 ± 0.2 in WT (P/Q; *t*-test: *p* = 0.0038); 1.3 ± 0.3 in SL/WT vs. 1.2 ± 0.2 in WT (N+R; *t*-test: *p* = 0.70).

To assess whether the FHM1 S218L mutation leads to increased AP-evoked Ca^2+^ influx and increased glutamate release at cortical pyramidal cell synapses, we studied excitatory neurotransmission in cortical neurons from neonatal SL/WT KI and WT mice grown in microculture. EPSCs were evoked in single cortical pyramidal cells by brief depolarizing voltage steps eliciting an AP in the unclamped axonal processes (Figure 1A, left inset). Tottene et al. (2009) have previously shown that P/Q-, N- and R-type Ca^2+^ channels cooperate in controlling glutamate release at cortical pyramidal cell autapses from WT mice (cf. superadditivity of the fractional EPSC inhibition by specific blockers of P/Q-, N- and R-type Ca^2+^ channels).

If the S218L mutation leads to increased AP-evoked Ca^2+^ influx through P/Q-type Ca^2+^ channels located at the active zones and, as a consequence, to increased probability of vesicle release, one predicts a larger AP-evoked EPSC amplitude and a larger contribution of P/Q-type Ca^2+^ channels to neurotransmission in SL/WT KI relative to WT mice, as previously found in RQ/RQ KI mice (Tottene et al., 2009). Indeed, the average EPSC amplitude was 1.8 times larger in SL/WT KI than in WT mice (Figure 1A), and a larger fraction of the EPSC was inhibited by the specific P/Q-type Ca^2+^ channel inhibitor ω-AgaIVA (Aga, 200 nM) in KI compared with WT neurons (Figure 1B), indicating a larger contribution of P/Q-type Ca^2+^ channels to synaptic transmission in SL/WT KI mice. The analysis of the absolute size of the component of the EPSC inhibited by this P/Q-type channel blocker (Tottene et al., 2009) reveals that the increased probability of release consequent to increased Ca^2+^ influx through P/Q-type Ca^2+^ channels completely accounts for the increased EPSC amplitude in SL/WT KI mice. In fact, the amplitude of the Aga-sensitive component was 2.1 times larger in KI than in WT mice, and the difference in size of this component in the two genotypes (1.5 nA) was similar to the difference in size of the corresponding total EPSC amplitude (1.6 nA; Figure 1C). On the other hand, the amplitude of the Aga-insensitive component was similar in KI and WT mice (Figure 1C). These findings are inconsistent with significant changes in the size of the readily releasable pool (RRP) of vesicles, at each synapse and/or in the number of synapses in SL/WT neurons, because change in the RRP or in the number of synapses would equally affect the Aga-sensitive and Aga-insensitive components of the EPSC (Tottene et al., 2009).

**Figure 2:**
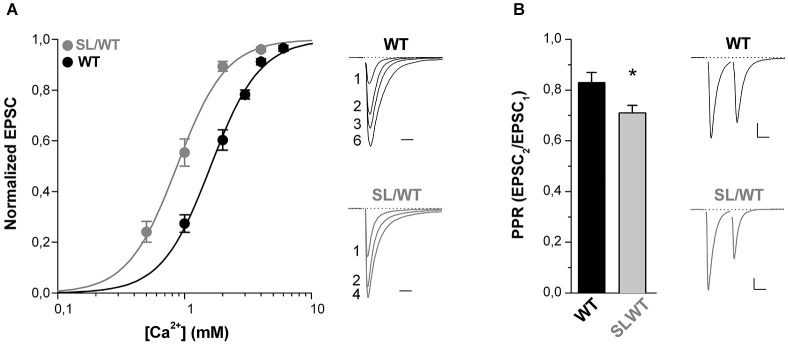
**The dependence of the EPSC on extracellular [Ca^2+^] is shifted to lower [Ca^2+^] and the paired-pulse ratio is decreased at cortical pyramidal cell autapses of SL/WT KI mice**. **(A)** Left panel: normalized EPSC amplitudes as a function of extracellular [Ca^2+^] evoked in cortical pyramidal cells in microculture (DIV 9–14) from WT (*n* = 7) and SL/WT KI (*n* = 11) mice. In each cell the EPSC data points were fitted according to the Hill equation: EPSC = EPSC_max_([Ca^2+^]^n^/(EC_50_)^n^+[Ca^2+^]^n^) and the EPSC amplitudes were then normalized to the EPSC_max_ obtained from the fit. The average normalized EPSC data points were fitted with EC_50_ = 0.87 mM, *n* = 2.2 for SL/WT KI mice and EC_50_ = 1.6 mM, *n* = 2.2 for WT mice. Right panel: representative normalized EPSC traces recorded at the indicated [Ca^2+^] (in mM) from a WT and a SL/WT neuron. Scale bar: 10 ms. **(B)** Paired-pulse ratios (PPR = EPSC2/EPSC1) evoked by two depolarizing stimuli at 50 Hz in cortical pyramidal cells in microculture (DIV 10–14). PPR values: 0.71 ± 0.03 (*n* = 43) in SL/WT and 0.83 ± 0.04 (*n* = 51) in WT neurons (*t*-test: *p* = 0.032). Right: representative EPSC traces evoked by two depolarizing stimuli at 50 Hz in a WT and a SL/WT pyramidal cell; scale bars 10 ms, 0.25 nA and 10 ms, 1 nA for WT and SL/WT, respectively.

Further supporting the conclusion of an increased AP-evoked Ca^2+^ influx through presynaptic mutant Ca_V_2.1 channels is the finding that in SL/WT KI mice the dependence of the EPSC on the extracellular concentration of Ca^2+^ ions [Ca^2+^]_out_ was shifted to lower [Ca^2+^]_out_ with unaltered cooperativity coefficient relative to WT (EC_50_: 0.90 ± 0.07 mM (*n* = 11) in SL/WT vs. 1.65 ± 0.10 mM (*n* = 7) in WT; *p* = 1.0 × 10^−5^) (Figure 2A), as previously found in RQ/RQ KI mice (Tottene et al., 2009).

Moreover, the finding that the paired-pulse ratio (PPR, the ratio between the amplitudes of the second and first EPSC elicited by two AP stimuli at 50 Hz) was lower in SL/WT KI than WT mice (Figure 2B) is consistent with and further supports the conclusion that the S218L mutation leads to an increased probability of vesicle release at individual cortical synapses.

The gain-of-function of both the evoked EPSC and the contribution of P/Q-type Ca^2+^ channels to neurotransmission as well as the Ca^2+^ dependence of the EPSC and the PPR are quantitatively similar in cortical pyramidal cell autapses of *heterozygous* SL/WT KI and *homozygous* RQ/RQ KI mice. In fact, according to Tottene et al. (2009), the evoked EPSC at cortical RQ/RQ KI pyramidal cell autapses is 1.7 times larger relative to WT, the fractional inhibition by Aga is 78 ± 3% and the EC50 of the Ca^2+^ dependence is 0.94 mM. Moreover, we measured a PPR of 0.63 ± 0.03 in response to 50 Hz paired-pulses at RQ/RQ cortical pyramidal cell autapses (*n* = 43). The similar increase in AP-evoked Ca^2+^ influx and probability of release in SL/WT and RQ/RQ KI mice correlates with the relatively small difference in gain-of-function of the Ca_V_2.1 current at low voltages in neurons from these mice (van den Maagdenberg et al., 2004, 2010).

**Figure 3:**
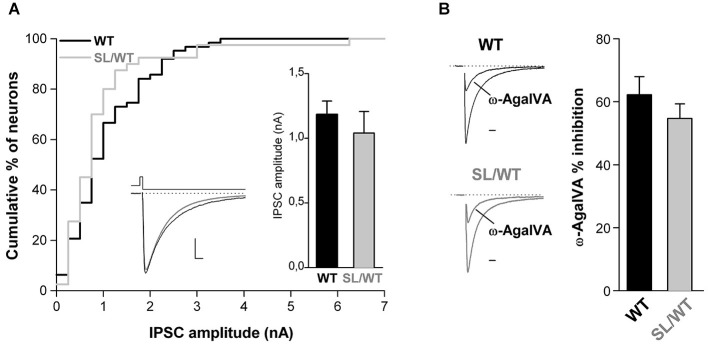
**Unaltered evoked IPSC amplitude and unaltered contribution of P/Q-type Ca^2+^ channels to inhibitory neurotransmission at cortical multipolar interneuron autapses of SL/WT KI mice. (A)** Cumulative distribution and average value (right inset) of IPSC amplitudes evoked in single WT and SL/WT cortical multipolar interneurons in microculture (DIV 10–14). IPSC amplitude: 1.0 ± 0.2 nA (*n* = 40) in SL/WT and 1.2 ± 0.1 nA (*n* = 63) in WT interneurons (*t*-test: *p* = 0.44). Left inset: representative IPSC traces from a WT (black) and SL/WT (gray) interneuron; scale bars: 10 ms, 250 pA. **(B)** Contribution of P/Q-type Ca^2+^ channels to inhibitory autaptic neurotransmission evaluated from the fraction of the evoked IPSC inhibited by Aga (200 nM, a saturating concentration since the IPSC was not further inhibited by 400 nM Aga, *n* = 4 not shown) in WT and SL/WT cortical multipolar interneurons in microculture (DIV 10–14): 55 ± 5% (*n* = 13) in SL/WT and 62 ± 6% (*n* = 17) in WT interneurons (*t*-test: *p* = 0.34). Representative IPSC traces before and after Aga (normalized to the control value) are shown on the left (scale bar: 10 ms).

To assess whether, as previously shown for the R192Q mutation (Tottene et al., 2009; Vecchia et al., 2014), the S218L mutation also does not affect inhibitory synaptic transmission at fast-spiking and other multipolar interneuron synapses, we studied inhibitory neurotransmission in cortical neurons from neonatal SL/WT and WT neurons grown in microculture. The recordings were performed on neurons characterized by irregular soma morphology with multiple asymmetrical processes emanating from it (cf. Figure 1 in Vecchia et al. (2014)), most of which (62–70%), but not all, had firing properties typical of fast-spiking interneurons (Vecchia et al., 2014). IPSCs were evoked in single cortical multipolar interneurons by brief depolarizing voltage steps (Figure 3A, left inset). Both the average amplitude and the fractional inhibition by Aga of the evoked IPSCs were similar in SL/WT and WT interneurons (Figure 3). From this we conclude that the S218L mutation, similar to the R192Q mutation, enhances excitatory neurotransmission at cortical pyramidal cell autapses but does not affect inhibitory neurotransmission at multipolar interneuron autapses, despite the large contribution of P/Q-type Ca^2+^channels in controlling GABA release at these synapses.

The unaltered inhibitory synaptic transmission and the similar increase in the strength of excitatory synaptic transmission in SL/WT and RQ/RQ KI mice, which is in good correlation with the similar facilitation of experimental CSD *in vivo* (van den Maagdenberg et al., 2010), are consistent with and support the conclusion that enhanced glutamate release at cortical pyramidal cell synapses may explain the facilitation of CSD in SL/WT KI mice, as directly shown in RQ/RQ KI mice (Tottene et al., 2009). Given the larger facilitation of CSD in SL/SL compared with SL/WT KI mice (cf. 67% vs. 38% reduction in CSD threshold and 140% vs. 42% increase in CSD velocity in SL/SL vs. SL/WT KI), that correlates with the larger gain-of-function of the Ca_V_2.1 current in SL/SL compared with SL/WT neurons (van den Maagdenberg et al., 2010), one would expect a larger increase in the strength of excitatory synaptic transmission at cortical pyramidal cell synapses in SL/SL compared with SL/WT KI mice.

**Figure 4:**
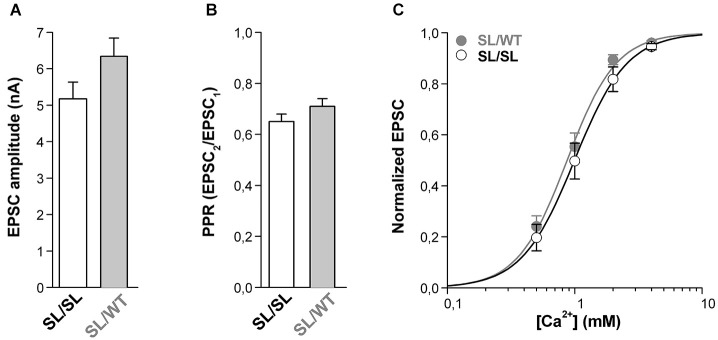
**Similar gain-of-function of excitatory neurotransmission at cortical pyramidal cell autapses of homozygous SL/SL and heterozygous SL/WT KI mice. (A)** Average value of EPSC amplitudes evoked in single SL/SL and SL/WT cortical pyramidal cells in microculture (DIV 10–14). EPSC amplitudes: 5.2 ± 0.5 nA in SL/SL (*n* = 46) and 6.3 ± 0.5 nA in SL/WT (*n* = 95) neurons (*t*-test: *p* = 0.14). **(B)** PPR evoked by two depolarizing stimuli at 50 Hz in cortical pyramidal cells in microculture (DIV 10–14) from SL/SL (*n* = 36) and SL/WT KI mice (*n* = 43). PPR: 0.65 ± 0.03 and 0.71 ± 0.03 in SL/SL and SL/WT neurons, respectively (*t*-test: *p* = 0.15). **(C)** Normalized EPSC amplitudes as a function of extracellular [Ca^2+^] evoked in cortical pyramidal cells in microculture (DIV 10–14) from SL/SL (*n* = 10) and SL/WT KI (*n* = 11) mice. The average normalized EPSC data points were fitted with EC_50_ = 0.99 mM, *n* = 2.1 for SL/SL KI mice and EC_50_ = 0.87 mM, *n* = 2.2 for SL/WT KI mice.

In contrast to this expectation, however, similar average EPSC amplitudes were evoked in SL/SL and SL/WT cortical pyramidal cells in microculture (Figure 4A). Moreover, the PPR was also similar (Figure 4B), suggesting a similar probability of release in neurons of the two genotypes. This is not due to near saturation of the presynaptic Ca^2+^ sensor at SL/WT autapses (cf. maximal value of evoked EPSC only 1.1 times larger than the EPSC at 2 mM Ca^2+^ in Figure 2A) because, even decreasing the [Ca^2+^]_out_ down to 0.5 mM did not reveal a larger EPSC in SL/SL compared to SL/WT KI mice (Figure 4C). Indeed, the evoked EPSC at SL/SL and SL/WT autapses showed a similar dependence on [Ca^2+^]_out_ (EC_50_ = 1.08 ± 0.14 mM for SL/SL KI (*n* = 10) and EC_50_ = 0.90 ± 0.07 mM for SL/WT KI (*n* = 11); *p* = 0.26) and, in a larger set of experiments, the ratio of the EPSCs evoked at 1 mM and 2 mM [Ca^2+^]_out_ was also similar in SL/SL and SL/WT KI mice (0.55 ± 0.04 in SL/SL (*n* = 21) and 0.61 ± 0.04 in SL/WT (*n* = 17); *p* = 0.23). If, as expected from the larger gain-of-function of the neuronal Ca_V_2.1 current in SL/SL relative to SL/WT KI mice (van den Maagdenberg et al., 2010), AP-evoked Ca^2+^ influx at the active zones is larger in SL/SL KI mice, and if no compensatory changes occur at SL/SL synapses (whereby the affinity of the presynaptic Ca^2+^ sensor is decreased and/or the capacity to rapidly buffer presynaptic Ca^2+^ is increased at the active zones of SL/SL compared with SL/WT KI mice), then the Ca^2+^ dependence of the EPSC at SL/SL autapses should be shifted to lower [Ca^2+^]_out_ relative to that at SL/WT KI autapses. The finding of a similar Ca^2+^ dependence of the EPSC in SL/SL and SL/WT KI mice is consistent with the existence of compensatory changes like those mentioned already and/or with a compensatory decrease in the number of functional Ca_V_2.1 channels at the active zones in SL/SL KI mice (see Section Discussion). Whatever the exact underlying compensatory mechanism, the similar gain-of-function of excitatory neurotransmission at cortical pyramidal cell synapses of SL/SL and SL/WT (and RQ/RQ) KI mice contrasts with the larger facilitation of CSD in SL/SL compared to SL/WT (and RQ/RQ) KI mice (van den Maagdenberg et al., 2010), and suggests that the further facilitation of CSD in SL/SL KI mice is due to gain-of-function of Ca_V_2.1-dependent mechanisms that are different from evoked glutamate release at cortical pyramidal cell synapses.

Notably, it has been recently shown that at Calyx of Held terminals a fraction of mutant Ca_V_2.1 channels is open at resting membrane potential in SL/SL KI but not RQ/RQ KI and WT mice; as a consequence, the intraterminal [Ca^2+^] and the frequency of mEPSCs were larger in SL/SL than WT calyces (Di Guilmi et al., 2014).

**Figure 5:**
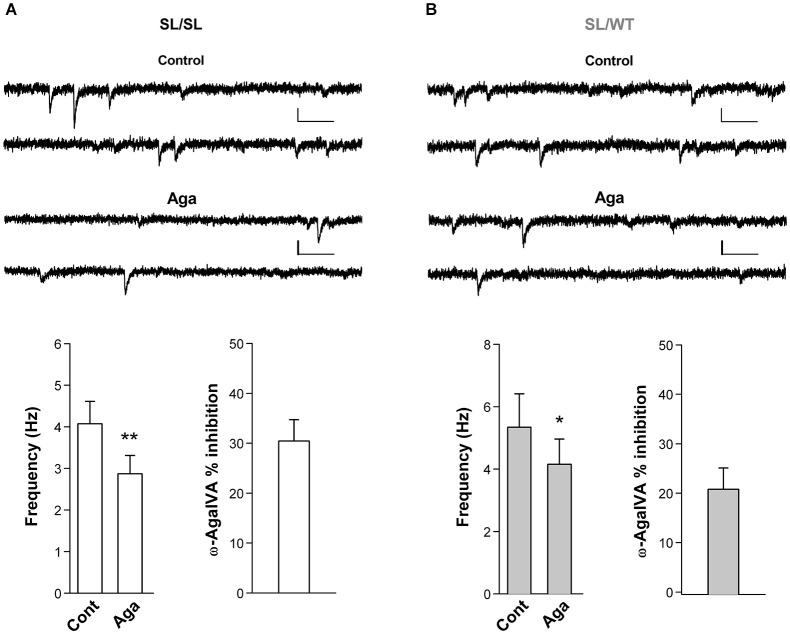
**Inhibition of P/Q-type Ca^2+^ channels reduces the frequency of miniature EPSCs in layer 2/3 pyramidal cells in acute cortical slices from SL/SL and SL/WT KI mice. (A)** Representative traces of mEPSCs recorded at −69 mV in a layer 2/3 pyramidal cell and average values of mEPSC frequency (*n* = 7) in control and after application of Aga (400 nM) in acute cortical slices from P16-18 SL/SL KI mice (Paired *t*-test: *p* = 0.0026); also shown is the average value of the percent inhibition of mEPSC frequency by the drug in each cell: 30 ± 4% (*n* = 7). Scale bars: 50 ms and 10 pA. **(B)** As in **(A)**, but recordings are from P16-17 SL/WT KI mice (*n* = 7) (Paired *t*-test: *p* = 0.012). Average value of the percent inhibition of mEPSC frequency by the drug in each cell: 21 ± 4%, *n* = 7. The difference in percent inhibition of mEPSC frequency by Aga in SL/SL and SL/WT neurons is not statistically significant (*p* = 0.14). Both amplitudes and frequencies of mEPSCs were similar in SL/SL and SL/WT KI mice: 8.3 ± 0.5 pA and 4.1 ± 0.5 Hz in SL/SL vs. 9.1 ± 0.7 pA and 5.3 ± 1.1 Hz in SL/WT; (*t*-test: *p* = 0.38 and 0.31, respectively).

To investigate whether a fraction of mutant Ca_V_2.1 channels open at resting membrane potential at glutamatergic synaptic terminals of cortical neurons from SL/SL and SL/WT KI mice, we recorded the frequency of mEPSCs in layer 2/3 pyramidal cells in acute cortical slices and measured its sensitivity to the specific P/Q-type Ca^2+^ channel inhibitor Aga. The frequency of mEPSCs was significantly reduced after application of Aga (400 nM) in both SL/SL (30 ± 4%) and SL/WT (21 ± 4%) cortical slices (Figure 5). In contrast, Aga did not affect mEPSC frequency in RQ/RQ slices (data not shown), in agreement with the similar frequency of mEPSCs reported in RQ/RQ KI and WT mice (Tottene et al., 2009). These data support the conclusion that a fraction of mutant Ca_V_2.1 channels open at resting membrane potential in cortical excitatory presynaptic terminals in both SL/SL and SL/WT KI mice, but not in RQ/RQ KI mice. Although there is a trend of a larger reduction of mEPSC frequency in SL/SL compared with SL/WT KI cortical pyramidal cells, the difference is not statistically significant.

## Discussion

Our analysis of excitatory and inhibitory synaptic transmission in microcultures of cortical neurons from S218L KI and WT mice revealed that the severer FHM1 S218L mutation, like previously found for the milder FHM1 R192Q mutation (Tottene et al., 2009; Vecchia et al., 2014), enhances excitatory neurotransmission at cortical pyramidal cell synapses without affecting inhibitory neurotransmission at multipolar interneuron synapses, despite the large contribution of P/Q-type Ca^2+^ channels in controlling GABA release at these synapses. This finding is consistent with the conclusion that the differential effect on cortical excitatory and inhibitory synaptic transmission is likely a common feature of FHM1 mutations, and further supports the idea that the neuronal circuits that dynamically maintain a tight balance between excitation and inhibition are likely altered in FHM1. A plausible working hypothesis is that dysregulation of the cortical excitatory-inhibitory balance in FHM1 may, under certain conditions (cf. migraine triggers), lead to hyperactivity of cortical circuits, mainly due to excessive recurrent excitation, that may create the conditions for the initiation of spontaneous CSDs (e.g., by increasing the extracellular K^+^ concentration, [K^+^]_e_, above a critical value; Vecchia and Pietrobon, 2012; Pietrobon and Moskowitz, 2014).

The increased strength of excitatory neurotransmission at cortical pyramidal cell autapses of S218L KI mice is due to increased probability of glutamate release consequent to increased AP-evoked Ca^2+^ influx through presynaptic P/Q-type Ca^2+^ channels. In fact, as previously shown for R192Q KI mice (Tottene et al., 2009): (i) the larger AP-evoked EPSC amplitude in S218L KI compared with WT mice was accompanied by a larger contribution of P/Q-type Ca^2+^ channels to synaptic transmission; (ii) the dependence of the EPSC on [Ca^2+^]_out_ was shifted to lower values; and (iii) the PPR was lower in S218L KI than in WT mice. The gain-of-function of both the evoked EPSC and the contribution of P/Q-type Ca^2+^ channels to neurotransmission as well as the Ca^2+^ dependence of the EPSC and the PPR at cortical pyramidal cell autapses of *heterozygous* SL/WT KI mice were quantitatively similar to those reported for *homozygous* RQ/RQ KI mice (Tottene et al., 2009), in good correlation with the similar gain-of-function of the neuronal P/Q-type Ca^2+^ current at low voltages and the similar facilitation of experimental CSD *in vivo* in SL/WT and RQ/RQ KI mice (van den Maagdenberg et al., 2010). This good correlation is consistent with and supports the conclusion that enhanced glutamate release at cortical pyramidal cell synapses may explain the facilitation of induction and propagation of CSD in SL/WT KI mice, as directly shown in RQ/RQ KI mice (Tottene et al., 2009).

However, in contrast with the larger neuronal P/Q-type Ca^2+^ current at low voltages and the larger facilitation of CSD in *homozygous* SL/SL compared with *heterozygous* SL/WT KI mice (van den Maagdenberg et al., 2010), the strength of excitatory transmission at cortical pyramidal cell autapses, the PPR and the Ca^2+^ dependence of the EPSC were all similar in SL/SL and SL/WT KI mice. These findings strongly suggest the existence of compensatory changes in SL/SL KI mice that prevent excessive glutamate release, and are consistent with e.g., a decreased affinity of the presynaptic Ca^2+^ sensor and/or a decrease in the number of functional Ca_V_2.1 channels at the active zones in SL/SL compared with SL/WT (and RQ/RQ) KI mice to compensate for the larger Ca^2+^ influx through open mutant Ca_V_2.1 channels. Evidence for the latter compensatory mechanism at Calyx of Held terminals of SL/SL KI mice is provided by the finding that, while the gain-of function of the Ca^2+^ current at low voltages is larger in Calyx terminals from SL/SL than RQ/RQ KI mice, the Ca^2+^ current at high voltages is reduced in SL/SL but unchanged in RQ/RQ KI compared with WT terminals (Inchauspe et al., 2010; Di Guilmi et al., 2014). This compensatory decrease in the number of functional Ca^2+^ channels may explain a decreased AP-evoked Ca^2+^ current in SL/SL KI compared with WT Calyx terminals (Di Guilmi et al., 2014). In contrast, AP-evoked Ca^2+^ influx is larger relative to WT at cortical pyramidal cell synapses (cf. left-shifted EPSC Ca^2+^ dependence) and cerebellar granule cell synapses (Adams et al., 2010) of SL/SL KI mice. The different alterations of AP-evoked Ca^2+^ influx at these different synapses may reflect the different durations of the AP (much shorter at Calyx terminals: Inchauspe et al., 2010; Di Guilmi et al., 2014) and/or different compensatory mechanisms.

Whatever the exact underlying compensatory mechanism, the similar gain-of-function of excitatory neurotransmission at cortical pyramidal cell synapses of SL/SL and SL/WT (and RQ/RQ) KI mice contrasts with the larger facilitation of experimental CSD in SL/SL compared with SL/WT (and RQ/RQ) KI mice (van den Maagdenberg et al., 2010), and suggests that the further facilitation of CSD in SL/SL KI mice is due to gain-of-function of Ca_V_2.1-dependent mechanisms different from evoked glutamate release at cortical pyramidal cell synapses. Gain-of-function of Ca_V_2.1-dependent mechanisms different from evoked-glutamate release should also underlie the unique cortical susceptibility to repetitive CSD events and the unique propensity of CSD to spread into subcortical structures (e.g., the hippocampus) observed in S218L (both larger in SL/SL than SL/WT) but not R192Q KI mice (van den Maagdenberg et al., 2010; Eikermann-Haerter et al., 2011).

A feature of cortical synaptic transmission in S218L KI mice not present in R192Q KI mice is the presence of a fraction of mutant Ca_V_2.1 channels that is open at resting potential in cortical excitatory synaptic terminals, as shown by the reduction in mEPSCs frequency produced by the specific P/Q-type Ca^2+^ channel blocker in S218L cortical slices (but not R192Q slices). As a trend, the fractional reduction was larger in SL/SL than SL/WT KI mice, but the difference did not reach statistical significance, possibly because even in SL/SL pyramidal cells only 30% of mEPSCs were Aga-sensitive. The compensatory changes that prevent a larger gain-of-function of evoked glutamate release in SL/SL compared with SL/WT KI mice may also reduce the difference in the fraction of Aga-sensitive mEPSCs between the two genotypes, and, in the case of a decreased affinity of presynaptic Ca^2+^ sensor(s), may account for the similar mEPSCs frequency in SL/SL and SL/WT pyramidal cells.

The fraction of mutant Ca_V_2.1 channels that are open at rest in cortical excitatory synaptic terminals is much smaller than that at Calyx terminals (cf. much larger, 76%, reduction of mEPSCs frequency by Aga at Calyx of Held synapses: Di Guilmi et al., 2014), probably as a consequence of expression of Ca_V_2.1 channels with a higher activation threshold (cf. half-voltage of activation of the WT P/Q-type Ca^2+^ current of −8 mV in cortical pyramidal cells (Tottene et al., 2009) and of −30 mV in calyces (Di Guilmi et al., 2014)), and a smaller shift to lower voltages produced by the S218L mutation (7 mV in pyramidal cells vs. 11 mV in calyces). As a consequence, one predicts a smaller rise of the intraterminal basal [Ca]_in_ in cortical excitatory terminals than measured in SL/SL Calyx terminals (Di Guilmi et al., 2014); this probably explains why the mechanism of facilitation of release (independent of Ca^2+^ influx and dependent on increased basal [Ca]_in_) that has been proposed to underlie the gain-of-function of AP-evoked glutamate release in Calyx (Di Guilmi et al., 2014), does not appear to contribute to the gain-of-function of excitatory transmission at SL/SL cortical pyramidal cell synapses. Nonetheless, since V resting of cortical pyramidal cells *in vivo* in awake animals and also in sleeping or anesthetized animals during the up-states is 10–20 mV more depolarized than that in acute cortical slices (Gentet et al., 2010; Mateo et al., 2011), the fraction of open mutant Ca_V_2.1 channels and the rise of basal [Ca]_in_ might be larger in S218L KI mice *in vivo*.

One can envisage several mechanisms by which Ca^2+^ influx at negative voltages sub-threshold for AP generation through presynaptic and postsynaptic mutant Ca_V_2.1 channels (Westenbroek et al., 1995) and increased basal [Ca]_in_ in cortical neurons may contribute to lower the threshold for CSD induction, increase the rate and extent of CSD spread and make the brain susceptible to repetitive CSD events. Available data suggest that a local regenerative [K^+^]_e_ increase, [K^+^]_e_-induced synaptic glutamate release following opening of Ca_V_2.1 channels and activation of NMDARs (and possibly postsynaptic Ca_V_2.1 channels) are key elements in the positive feedback cycle that ignites CSD (Pietrobon and Moskowitz, 2014). This model predicts a lower threshold for CSD initiation and a higher rate of CSD propagation (mediated by diffusion of K^+^: Pietrobon and Moskowitz, 2014) in SL/SL than in SL/WT KI mice, even with similar AP-evoked glutamate release in the two genotypes, if a larger fraction of mutant Ca_V_2.1 channels opens at sub-threshold depolarizations induced by local [K^+^]_e_ rise in SL/SL KI mice. Moreover, Ca_V_2.1-dependent release of other neurotransmitters or neuromodulators (e.g., CGRP: Tozzi et al., 2012) may be enhanced in SL/SL KI mice relative to SL/WT and RQ/RQ KI mice and might contribute to the further facilitation of CSD in homozygous S218L KI mice. It is also possible that only in SL/SL neurons [Ca]_in_-activated cationic channels contribute to the net self-sustaining inward current necessary to initiate the positive feedback cycle that ignites CSD (Somjen et al., 2009; Pietrobon and Moskowitz, 2014). Finally, a constant basal Ca^2+^ influx would put a metabolic burden on cortical neurons of S218L (but not R192Q) KI mice that may prolong the transient hypoxia and slow the recovery of cerebral flow after CSD (Pietrobon and Moskowitz, 2014) and in general make the recovery from CSD more difficult. Since another peculiarity of mutant S218L Ca^2+^ channels, besides the particularly low threshold of activation, is the incomplete inactivation during prolonged depolarizations (Tottene et al., 2005), a larger increase of [Ca]_in_ during CSD in cortical neurons may also contribute to a slower recovery from CSD in S218L KI mice. A slower recovery from CSD could provide a plausible explanation for the unique susceptibility to recurrent CSDs (van den Maagdenberg et al., 2010) and the unique propensity of CSD to spread into subcortical structures such as hippocampus and thalamus as well as the more severe motor deficits after CSD (Eikermann-Haerter et al., 2009, 2011) as observed in S218L KI mice. It has also been proposed that these unique features conferred by the S218L mutation may underlie the severe and clinically broad phenotype that is seen in patients carrying the S218L but not in patients carrying the R192Q mutation (Eikermann-Haerter et al., 2009, 2011; van den Maagdenberg et al., 2010).

## Author and contributions

Dania Vecchia performed the experiments, analysed the data, discussed findings and interpretation of the data and contributed to drafting the manuscript.

Angelita Tottene contributed to the acquisition, analysis and discussion of the data and revised critically the manuscript.

Arn M.J.M. van den Maagdenberg contributed to the discussion and interpretation of the data, provided reagents and analytic tools, and revised critically the manuscript.

Daniela Pietrobon conceived and designed the study, discussed findings and interpretation of the data and wrote the manuscript.

## Conflict of interest statement

The authors declare that the research was conducted in the absence of any commercial or financial relationships that could be construed as a potential conflict of interest.
